# The Perception of Urban Forests in Post-Mining Areas: A Case Study of Sosnowiec-Poland

**DOI:** 10.3390/ijerph19073852

**Published:** 2022-03-24

**Authors:** Robert Krzysztofik, Oimahmad Rahmonov, Iwona Kantor-Pietraga, Weronika Dragan

**Affiliations:** 1 Institute of Social and Economic Geography and Spatial Management, Faculty of Natural Sciences, University of Silesia, Będzińska 60, 41-200 Sosnowiec, Poland; iwona.kantor-pietraga@us.edu.pl (I.K.-P.); weronika.dragan@us.edu.pl (W.D.); 2 Institute of Earth Sciences, Faculty of Natural Sciences, University of Silesia, Będzińska 60, 41-200 Sosnowiec, Poland; oimahmad.rahmonov@us.edu.pl

**Keywords:** urban forest, post-mining city, social perception, brownfields management, Poland

## Abstract

Sustainable development policy emphasizes, among other things, the role of green areas in urban space. This remark applies in particular to post-industrial and post-mining cities. One of the elements of shaping the sustainable development of post-mining cities is that forests are often anthropogenic forest ecosystems growing in previously mining areas, one of the most characteristic elements of their spatial development. This article examines the role of urban forests in the post-mining area in Sosnowiec, located in the core of the Katowice conurbation in southern Poland. This article aimed to show the social perception of forests in post-mining areas among the local community and the features of urban forests. The social dimension of the interaction between humans and the environment is related to the issue of urban planning. Research was implemented based on quantitative, qualitative (CATI survey), and cartographic methods. The results indicate the significant role of forests in post-mining areas depending on their location in the settlement areas in a post-industrial city. The research emphasizes that residents perceive forests in post-mining areas of cities as an essential and expected recreational space. Notably, half of them do not see any threats therein. It is also expected that these areas will be better developed for recreational purposes in the future.

## 1. Introduction

Nowadays, the concept of sustainable urban development is expected to feature in the design and development of urban areas. However, social expectations for implementing this idea are not always visible locally. Moreover, the idea of sustainability may be viewed differently by residents of the same city, e.g., as well-being created by economic needs or as well-being created by the need for self-realization and leisure activities [1,2,3,4]. These expectations even diverge in post-industrial cities or shrinking cities [5,6,7]. This is all the more surprising since, given the problems and challenges of such cities, the concept for developing them should firstly be coherent and, secondly, strive for balance. The spatial and functional elements that should be essential attributes of the idea of sustainability in this type of city include the development of green areas (mainly urban forests and parks). In many cities, however, the scarcity of green spaces as a type of space that can indirectly reinforce the idea of local urban sustainability remains problematic. This is especially true in those centers where advanced urbanization exacerbates the scarcity of wooded areas [8,9].

However, economic transformation provides an opportunity to increase the proportion of green space, which has resulted in some areas previously occupied by industry or mining losing their former functions. The resulting derelict land, developed as new parks or recreational areas, competes with the vision of being used once more for construction [10]. Post-mining towns are a particular case in this respect. This uniqueness stems from certain restrictions on implementing new industrial or residential functions in post-mining areas. Each case of this type increases the opportunities for green areas (anthropogenic forests) to be developed. By anthropogenic forests, we mean an urban forest that emerges spontaneously or planned on the land formerly used by the mining. Such areas may be created both through planned afforestation and natural succession. An urban post-mining forest, as with any such green area, undoubtedly plays an important ecosystem role in the urban environment *per se*, although its social role is also significant [11,12,13].

This article aimed to show the social perception of forests in post-mining areas among the local community and the features of urban forests in a big city—Sosnowiec in the Katowice conurbation (Poland). The analyzed city, on the one hand, stands at a crucial juncture in its economic transformation and, on the other, aims to implement the idea of sustainable development.

The authors attempt to answer the following question: can urban forests in post-mining areas benefit society similarly to parks? This question is important because, from the perceptual point of view, post-mining forests constitute an intermediate element between brownfield sites (which have a rather negative image) and city parks commonly treated as the expected form of land use planning in urban space.

Undoubtedly, an essential element of this research was its analysis of local community opinions as both a social voice within the idea of governance and essential guidance in urban policy and the further directions for spatial development.

## 2. Research Overview

The directions of land use in post-mining towns and the social dimension of functional transformations have been researched extensively in several studies. However, the scientific output devoted to forests in post-mining areas is markedly narrower in scope. At this point, it is necessary to emphasize four strands of research that appear to be crucial from the perspective of the research problem and this article’s aim.

The first strand of research concerns urban forest biocoenosis and its role in how the urban ecosystem functions. Urban forests are a particular ecological system artificially created in urban areas, and socio-economic factors often condition how they function. Urban forests are often identified as urban parks [14,15], and have many social benefits: environmental, natural, social, ecological, and ecosystem, and also improving the environmental and sanitary conditions of the city [16,17,18,19,20,21]. Forest ecosystems in the city or on its edge undoubtedly impact reducing noise. Their root systems can reduce vibrations or absorb and reflect sound waves [22]. Increasing the attractiveness of a place to live affects the real estate value [23] within a city. One of the essential roles of urban forest ecosystems is their ecological benefits [24,25] in shaping biodiversity or as an element of an ecological corridor [26].

The second strand of research concerns the development of sustainable cities (cf. [23,27,28,29]). The key problems, in this case, concern the essence of sustainable development in post-industrial and post-mining cities. These cities have a kind of carte blanche as to their ability to set the direction of future change (cf. [30,31,32]). Research within this discourse indicates the need to align the development of post-industrial cities with new growth opportunities and points to the constraints that accompany these goals, such as environmental degradation. An entirely separate issue within this research strand is the challenges posed to sustainable urban development by protecting the climate and implementing adaptive policies (cf. [33,34,35]). Also visible in the Sosnowiec region is the idea of a regionally understood just transition, focused on meeting local communities’ economic and social needs due to the decommissioning of coal mining [36,37].

The third strand of research related to this article’s objective is the governance of urban shrinkage (cf. [38,39,40,41]). This strand strongly relates to the previous one, developing sustainable cities. However, it focuses more on managing a post-industrial city undergoing the urban shrinkage stage or stuck in it for a long time. In this research strand, the issue of urban development is seen as more bottom-up.

On the other hand, the spatial-functional problems of the city are one of the fundamental challenges of local politics [5,42,43]. From the point of view of forests in post-mining areas and or more broadly understood green areas in the broader sense, an important issue arises. It concerns the role and benefits of these areas in the process of leveling spatial and socio-demographic problems—green areas as a factor in the attractiveness of a place of residence [44,45,46,47,48]. This problem is all the more important because, in a situation where cities compete for economic investments, the residential functions represent the only possibility for the further development of the district or town.

The fourth strand of research we refer to in this article relates to the dilemmas of brownfield development in urban policy and local governance (cf. [6,34,49,50]). Brownfield sites and derelict areas are a natural and important cognate for forest communities in post-mining areas. Since every forest that has grown on a post-mining site occupies an area that was historically a brownfield site, the question of how to develop and manage such areas is fundamental [51,52]. The opinion of the city residents on the needs related to rest, recreation and access to green areas are particularly essential here. Inhabitants are one of the basic stakeholders of urban policy and governance modes. Research on the directions of developing brownfield sites, their location, structures, and urban (or regional) governance relating both to the directions of their transformation and the co-participation of different stakeholder groups within them is therefore important here [50,53,54]. Additionally, issues related to the environmental conditions of brownfield sites in terms of their possible development, such as the nature of the substrate, possible contamination, and environmentally valuable areas and ecological corridors, are also significant [47,55,56,57]. The issue of urban forests in post-mining areas is related to all four of the research strands presented above [5,39,58,59].

## 3. Sources and Methods

Several sources were used in the research on forest communities in post-mining areas. The first were cartographic sources, historical and contemporary, indicating the extent of forests in post-mining areas within the city’s borders being analyzed.

A detailed inventory for statistical purposes was made on the basis of an orthophoto map (the determination of the contemporary extent of the tree stand boundary, which is burdened with an error resulting from the inability to determine an exact position of the boundary, but only an approximate one—due to taking a photo of the area at an angle, and gradual thinning of the stand—blurring a sharp border, due to the succession of vegetation).

The existing databases are incomplete and require updating (BDOT and UM data); moreover, they present the extent of the trees in a generalization. Smaller trees, however, within the 0.1 ha criterion, between dense buildings, including within properties, were not taken into account.

In addition, to correctly classify the tree cover (park, post-mining forest, other wooded areas), the content of archival topographical maps from 1926, 1959, 1980, and 1990 was used [60].

The forests include narrow roads and paths, ruins of old buildings, clearings, small watercourses, and water reservoirs. In order to estimate the stand compactness, the Braun–Blanquet [61] scale was used, in which the following categories were applied: degree 5—species covering the 75–100% of the area degree 4—species covering the 50–75% of the area, and degree 3—species covering the 25–50% of the area.

The current extent of forest areas was also verified based on Google Earth data and, during the field experiment, research using a DJI Phantom 4 Advanced drone (SZ DJI Technology, Ltd., Shenzhen, China). The drone analyzed forests in hard-to-reach places and confirmed tree cover depicted by maps and satellite data. Cartographic methods were also used in this paper. Based on QGIS software, a detailed map of forests in post-mining areas in Sosnowiec was produced. Field studies also constituted the basis for learning about the species diversity of the forest biocoenosis.

Another essential source of analysis was found in the planning and strategic documents of the City of Sosnowiec: Local Program for the Revitalization of the City of Sosnowiec for 2016–2023 [62]; Strategy for the development of Sosnowiec until 2020 [63]; Sosnowiec—Master Plan—Study of the Conditions and Directions for Spatial Development [64]. These documents made it possible to determine the formal status of the areas being analyzed, the planned directions for their development, and the existence of potential future planning conflicts. This essential element of the research indicates a certain outline of the trends and roles of forests in post-mining areas in Sosnowiec. Although it provides a basis for planning, it is not the only attribute that shapes geographical reality. Poland’s spatial planning system allows previous arrangements to be modified quite flexibly, especially in those cases where there is what is deemed a ‘socially justified purpose or benefit’ [14]. Therefore, we were also interested in the actual activities of the local government and economic entrepreneurs and those related to the investment policy in the area of forests on post-mining land or in their immediate vicinity. This was also supported by analysis of the information available in the Public Information Bulletin (B.I.P.) system and information obtained from the media, including social media (www.sosnowiec.gazeta.pl; www.twojezaglebie.com; www.dziennikzachodni.pl and others) (accessed on 20 November 2021).

The social dimension of the research was implemented based on a survey developed using the computer-assisted telephone interviewing (CATI) method. The survey was carried out in October 2020. Basic statistical information on the distribution of the sample by gender and age is presented in Table 1.

The survey was designed to identify local community opinions on the perception of forests in the post-mining area and their role in selected aspects of personal and social life. Only surveys in which the respondents expressed their opinions on post-mining forests and parks were included in the research. Respondents were asked five questions:

1. How often do you visit a forest or a park in the city of Sosnowiec?
2. Should a green area in your area be a more accessible forest or park?
3. Should this area be better managed (alleys, lighting, recreation)?
4. Do you appreciate this area’s natural diversity?
5. What deters you from wandering in a forest or park in the city? (See more in Appendix A)

The chi-square test of independence was used as the basis for the research on associations in the data on the analyzed features. The calculations were prepared based on the Statistica 13 software package (TIBCO Software Inc., Palo Alto, CA, USA).

## 4. Results

### 4.1. Features of Urban Forests

#### 4.1.1. Diversity of Forests in Urbanized and Post-Mining Areas in Katowice Conurbation and Sosnowiec

Despite intense urbanization and industrialization, forest areas are an essential element of the landscape of the cities of the Katowice conurbation. From the perspective of the sustainability of forest areas, two main types of forest can be distinguished here: semi-natural forest and anthropogenic forest created in spaces that earlier performed other functions. According to A. Storm [66], “industrial nature” arose due to plant succession to brownfield sites, the result of which may be a forest that is a habitat rich in various species. Such forests include forests in areas previously used for mining. These forests in post-mining areas can also be classified as a type that Henne [67] referred to as ‘new wilderness’ and which Bonthoux et al. [68] called ‘an abandoned site with spontaneous vegetation or wild-grown vegetation (i.e., [59]).

The vast majority of the contemporary flora in the Katowice conurbation diverges from potential vegetation, indicating how the natural environment has been transformed under human activity. Pine forests were developed on sandy post-glacial formations in places with standing water previously overgrown with alder swamps. The natural vegetation was destroyed and transformed, and as the development of mining entailed the need for wood, it was necessary to cut down trees. In natural deciduous forests, monocultures of fast-growing pine were planted [69].

Opencast mining and dumps of post-mining waste have led to the destruction of soil and plant cover. One of the consequences of human activity in this area is ‘post-mining forests.’ A post-mining forest is a type of urban forest. It can be distinguished both from a park (economic function) and from an urban forest that has grown continuously in a particular place for centuries (natural conditions). Forests in post-mining areas are often secondary to previously existing natural structures (e.g., they are created on meadows where mining later took place).

**Figure 1:**
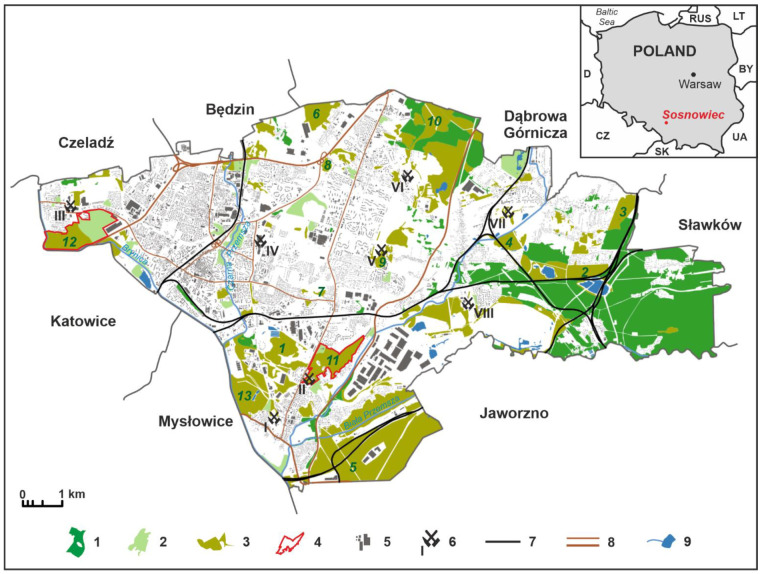
Forested areas in city of Sosnowiec, 2021. Explanations: 1—forests, 2—parks, 3—forests on the brownfield sites, 4—forest described in the article, 5—built-up areas, 6—closed coal mines, 7—main railways, 8—expressways, 9—rivers, and reservoirs. Names of highlighted postmining forests: 1—Bergi; 2—Bory; 3—Dorota; 4—Hałda Feliks/Zawodzie; 5—Jęzor Południe; 6—Józefów; 7—Kamionka—Dańdówka; 8 Kamionka—Mec; 9—Klimontów (Kopalnia); 10—Las Zagórski; 11—Lasek; 12—Milowice; 13—Rybaczówka. Names and localisation of closed coal-mines: I—Niwka-Modrzejów (Modrzejów), II—Niwka-Modrzejów (Niwka), III—Saturn (Milowice), IV—Sosnowiec (Sielec), V—Porąbka-Klimontów (Klimontów), VI—Porąbka—Klimontów (Zagórze), VII—Kazimierz-Juliusz (Kazimierz), VIII—Kazimierz—Juliusz (Juliusz). Source: by authors.

In conclusion, the locations of this type of forest are post-mining brownfield sites as a form of land use (former mining opencasts, slag heaps, mining buildings, mining and transport infrastructure within the mining areas, illegal mining areas from the interwar period). The distribution of the types of forest complexes in Sosnowiec, located in the core of the Katowice conurbation, is presented in Figure 1.

This area of undoubted value also plays a crucial role in the region’s sustainable development, especially as it relates to post-industrial, post-mining towns strongly affected by demographic and social problems. Although urban forests have always been an essential element of master plans for individual spatial development plans in the region, there have been different approaches to post-mining forests. However, the following issues draw attention in regional planning for forests in post-mining areas:

− the discrepancies between forest areas delimited in master plans and the actual extent of forests,
− inconsistent trends in managing forest areas created as a result of succession in post-mining areas (with part of this area having the status of a forest, part the status of wasteland, and part the status of land with other uses),
− the intense focus of municipal strategies and master plans towards the social role of parks, with the visible marginalization of forests
− weak promotion of valuable natural fragments of forests and their biodiversity.

The discord between the valuable natural merits of forest complexes created on post-mining areas and the issue of planning and managing them is also reflected in how they are perceived socially. This issue, which is discussed further in this article, is essential because of the severe degradation of the region’s environment and the challenges posed by the transformation from a mining region to a sustainable region. The city of Sosnowiec is a typical example of these challenges and problems.

The features of forests in the post-mining areas in Sosnowiec are presented in Table 2 based on 13 examples, marked with numbers in Figure 1. The list includes forests in areas with various forms of previous mining activity.

The stand includes both native species (*Acer platanoides, A. pseudoplatanus, Alnus glutinosa, Betula pendula, Carpinus betulus, Fraxinus excelsior, Populus tremula, Fagus sylvatica, Quercus robur, Ulmus laevis, and Pinus sylvestris*) and alien species (*Acer negundo, A. saccharinum, Quercus rubra, Robinia pseudoacacia, Pinus nigra*). In older forests (50 years) on brownfield sites, a shrub layer and loose undergrowth are formed with the participation of acidophilic species. Similar species composition was found in other post-industrial areas of southern Poland [70].

Poland’s potential vegetation map shows that the area was overgrown with fertile deciduous forests such as *Tilio-Carpinetum*, *Dentario enneaphylli-Fagetum, Querco-Pinetum* on hills, and *Fraxino-Alnetum* in wetlands and sandy surfaces covered mainly by *Leucobryo-Pinetum* [71].

The vast majority of forest communities growing in post-mining areas are anthropogenic. They were created due to the conscious introduction of woody species resistant to extreme habitats. In terms of species composition, the analyzed forests are very poor due to the age of the stand and habitat conditions, which are often extreme here. The biodiversity of post-mining forests is undoubtedly enhanced by wetlands and aquatic areas [72] within the study sites. In terms of species composition and the vertical structure, the studied forest phytocenoses differ in post-mining areas associated with limestone mines and coal mines.

Within the forests summarized in Table 2, the dominant forests are those aged about 30 and 50 years and that began to grow in the mid-20th century. Their age is closely related to two phases of deindustrialization in the mining sector [73].

Regardless of their origin (natural or anthropogenic), the urban forests in Sosnowiec differ in many respects (stand age, species composition, area, location). An urban forest is a forest comprising woody and shrub vegetation that grows directly in the vicinity of human settlements, and may arise both due to natural succession and artificially introducing species. Unlike an urban park, urban forest phytocoenoses can be inherited from the remains of primeval forest ecosystems. There are all kinds of amenities in city parks (benches, swimming pools, paved paths), and there are no such elements in urban forests. The functioning of ecosystems here is under the influence of natural processes. However, they provide similar ecosystem services, which can be considered in four categories (provisioning, regulating, supporting, and cultural) distinguished by De Groot et al. [74] and Millenium Ecosystem Assessment (MEA) [75].

#### 4.1.2. Forests in the Post-Mining Area—A Problem or an Opportunity for Town Planning in Sosnowiec

Forest ecosystems in the post-mining area in Sosnowiec have developed since the 1930s–1940s. At the end of 2021, their area was 1354 ha. The remaining forests and trees area is 2761 ha, so it is only about twice as large as the area of post-mining forests. Both types of forests cover 45.2% of the city, 14.9% of which are post-mining forests. The parks cover 207 ha, which is 2.3% of the area of Sosnowiec. Post-mining forests connected with the economic consequences of the post-socialist transformation (period: 1989–2021) occupy about 25% of this kind of green area. Only about 20% of the post-mining forests from the post-socialist period are forests created by planned planting. This mainly concerns coal heaps. Most of the forests in Sosnowiec’s post-mining areas were created due to spontaneous vegetation succession. This feature means that in many cases, they are treated by residents as a “natural forest,” as ‘green areas.’ By contrast, areas that humans create are considered parks or green but forestless wastelands. However, the fall of socialism and the negative consequences of the socio-economic transformation (depopulation, deindustrialization) brought unexpected changes compared to the preceding socialist period. The human penetration of forests in post-mining areas decreased. From about 1995–2000 onwards, large mammals almost absent before 1989 (wild boars, foxes, beavers, and elk) started to be recorded in all larger forest complexes. This phenomenon is described in Rahmonov et al. [19].

In this context, one should refer to the question posed in the research problem: what social role can forests play in post-mining areas in a post-industrial city in the first half of the 21st century? Another query also emerges from this question: can these forests perform recreational functions similar to those of parks? The sources and methods used in the research made it possible to answer the above questions.

### 4.2. Results of Empirical Research

#### 4.2.1. Forests in Post-Mining Areas in Documents

The fundamental problem that had to be solved at the research stage based on planning analyses was an attempt to confront the geographical reality (a wooded area visible in space, located in an area previously used for mining) with the typology of the areas presented in documents [76,77]. Firstly, forests in post-mining areas are marked on the Master Plan map as wastelands, building areas, forest areas, parks, industrial areas, green areas, or other areas. However, it should be noted here that the geographical reality has been significantly updated since the previous document. Opania and Szaton [78] also pointed this issue out. In their draft linear concept of landscape protection in Sosnowiec and neighboring cities, they included forests in post-mining areas in the following categories: forest, wooded areas that are neither forest nor park, park, low greenery (ordered and unordered), degraded areas, areas “with anthropogenically transformed relief,” and unreclaimed areas with visible degradation of natural environment components. However, in the map presented by the authors and the Sosnowiec—Master Plan—Study of the Conditions and Directions for Spatial Development [64], the indicated land types do not only relate to forests in post-mining areas. On the other hand, not all forests in post-mining areas have been classified into the land use types listed here in the publications above. A difficult research problem is also posed by post-mining areas used differently for a short period. However, these areas would eventually transform into a forest due to succession (agricultural land, allotment plots, and orchards or unrealized economic and municipal investments).

However, the above differentiation is most emphasized because there is a strong contrast between the selected fragments of postmining forests cut down in recent years (according to the Master Plan, these were areas of economic and residential investment). This applies to the Niwka district and the former sand mine, which is a protected area—the forest and peat bog ‘Bory’ in the city’s eastern part.

Although such a typologically diverse presentation of these areas may be problematic from the cognitive point of view, this is explained by the essence of the Master Plan, which is also supposed to indicate the possibilities of city development through changes in the current land use. The significant shortcoming of the Master Plan’s provisions for the geographical reality is the limited scale of implementation of some of the undertakings proposed. This is especially true of ventures to increase inhabitants’ access to these areas while, at the same time, leaving them as forest areas rather than park areas (occasional cleaning, creation of small infrastructure for walkers and cyclists). This process is visible in Milowice, Kazimierz Górniczy and Niwka districts. The divergence above in functions and actual management is often the result of a complicated ownership structure. Forests in Sosnowiec’s post-mining areas belong to the state-owned Mine Restructuring Company (S.R.K.), Sosnowiec commune, and State Forests. Some fragments belong to private owners, companies, or their legal status is not fully regulated.

The conditions mentioned above significantly limit the possibilities of implementing a coherent policy of developing forests in post-mining areas as an essential component of the city’s natural system.

In addition, key elements conditioning urban policy towards forests in post-mining areas are:

− pressure to develop economic and residential investments,
− diverse natural and geological conditions,
− specific features of individual post-mining areas (e.g., pit, slag heap, leveled area, an area requiring decontamination),
− location of a given forest complex,

In the most general terms, urban policy is made visible in four lines of action:

(a) renaturalization and reforestation,
(b) reindustrialization,
(c) functional pausing,
(d) parkification (cf. also [79]).

In the first of these directions, the activities of municipal authorities are aimed at preserving the forest, and in justified cases, even its protection. In Sosnowiec, the area of the former sand mine (“Bory”) has been under protection. The mine has been transformed into a fen with protected plants in the last few decades. It should be noted, however, that reforestation mainly concerns highly degraded areas, where any construction or infrastructure would be exposed to damage. It is also relatively easier to adopt this course of action in areas where the forest has grown since the 1970s.

The most common model for transforming post-mining brownfield sites is re-industrialization [73]. This applies primarily to areas in the most urbanized western part of the city, the relatively least degraded areas, and those with good transport connections. The existing tree canopies here have a chance of survival, as long as they are not located within newly constructed buildings or their accompanying infrastructure.

The third type of urban policy can be termed a functional pause. In this case, there are discrepancies between the provisions of the Master [64] and reality. However, there is the question of the potential future use of these areas. A good example from 2021 is the deforestation proposed by the municipal authorities in the eastern part of the city, which aims to free up this area for housing. Public opposition to the tree felling was summed up by a representative of the municipal authorities, who stated that “these houses are being built on land that is formally grassland”. This is, after all, what is stated in the Master Plan. It also seems that it is easier to obtain decisions on felling trees in areas not defined as forests in the Master Plan.

The fourth type of urban policy focuses on managing the forest in the post-mining area in the direction of park functions. This transformation model was already present in Sosnowiec before the Second World War, and was developed in the socialist period. As a rule, park spaces occupied part of a more extensive forest in the post-mining area. They were located close to residential areas and enjoyed good transport connections. This trend continues today (Millennium Park). Although this development direction concerns a relatively small area of post-mining forests, it enjoys the most significant interest among the residents due to its social dimension and the media-friendly nature of the topic. The qualitative research presented in the next part of this article adds weight to this thesis.

#### 4.2.2. Forests in Post-Mining Areas, in the Opinion of Residents

A CATI survey (encompassing the whole city of Sosnowiec) was conducted with a group of 300 adult residents of Sosnowiec. At the outset, it should be noted that the results of the chi-square test of independence (with α = 0.05) indicated that several of the analyzed features were statistically significant. The survey concerned both respondents’ age and sex with the selected opinions and associations regarding opinions on two different issues. Detailed test results are presented in Appendix B. In the following list, statistical significance is indicated in the discussion of individual sub-outcomes.

As mentioned, the questionnaire was addressed to inhabitants of different parts of the city in proportion to the population of particular districts.

The period during which the study was carried out, during the COVID-19 pandemic, particularly demonstrated the importance of green spaces in a highly urbanized and air polluted area [80,81]. Forests became an important place for residents to visit, and for the 13.3% of respondents who visited the forest daily, they were as important as many other activities performed each day. For 53% of respondents, green areas were places to spend leisure time once a week. It is worth noting that the need to relax in the forest increased with the age of the respondents. In the 18–34 age group, the proportion of respondents visiting a forest once a week was 7.3% for women and 5.7% for men. However, among residents aged 55 and over, this increased to 11.7% and 11.3%, respectively (Table 3).

As already mentioned, one of the essential elements of green space development policy is the issue of possible conversion. Although most residents, especially women (Table 4), favor this conversion, it should be noted that the group of people supporting the status quo is also significant. The functions of the forest are essential to the elderly. This is probably because more than 50 different outdoor recreation spaces have been created in the city; some of the green areas are used mainly by residents in the younger age brackets. The forest can provide an alternative to places with extensive infrastructure for relaxation and recreation. The fact that this study was conducted at the height of the COVID-19 pandemic was also significant [80]. For some of the older residents, the forest could have been a place that met the criteria of isolation and, at the same time, the need for physical activity, and did so better than the sometimes crowded parks.

A natural consequence of the opinion on whether forests should be converted into parks was the answers given by Sosnowiec inhabitants concerning land use. Such a need was indicated by two-thirds of those interviewed. Generally, this was important for people aged 55 and over, and more so for women (Table 5). Interestingly, in 16 questionnaires concerning the need to preserve the forest, the need for more advanced management was also indicated.

The importance of the relationship between the frequency of visits to the forest and the need to increase its availability [χ^2^ (5, N = 300) = 37.5, *p* = 0.00005, ΦC = 0.250] should also be pointed out. An exception is the group of people rarely visiting the green areas—notably, most respondents of all ages and sex.

Inhabitants of Sosnowiec also indicate the need for better management of green areas. The relationship with the issue of the frequency of visits to green areas is meaningful [χ^2^ (5, N = 300) = 31.1, *p* = 0.00057, ΦC = 0.228].

While the average city dweller is not an expert on biodiversity, they see it personally. Undoubtedly, the most polarized opinion concerned the natural value of the forests in Sosnowiec (Table 6). The natural diversity of the forest is valued by as many as 82.7% of inhabitants. Interestingly, this diversity is often not very distinct in the sense of species. It is dominated by birch and *Robinia* forests, and in the eastern and southern part of the city, also by pine forests. The value for the inhabitants seems to lie more in the vegetation layers and the density of trees, which is higher than in parks. Biodiversity connects respondents of both genders, with a slight predominance of women aged 55+. The statistical significance of this feature is associated—as in the previous cases—with the frequency of visits to forests within the city [χ^2^ (5, N = 300) = 42.6, *p* = 0.00001, ΦC = 0.267].

Undoubtedly, an essential element of the survey was the question about the real problems related to how Sosnowiec’s forests are perceived and used (Table 7). Of the problems listed, two stand out as being the most important: the threat of a tick bite (24.7% of respondents), which in most cases is connected with contracting Lyme disease or meningitis, and the distance of the forest from the place of residence (14.3% of respondents). Ticks are of more significant concern in men aged 18–34 and 35–54. In the age group 55 and more, the group of women stands out. In the case of wild animals, in two age groups 18–34 and 55 years and above, the answers were the same. Only in the group of middle-aged respondents do men prevail.

However, from the point of view of the chi-square test of independence, the awareness of this risk versus the age of the inhabitants is statistically significant [χ^2^ (5, N = 300) = 6.60, *p* = 0.036, ΦC = 0.148]. In this case, the relationship is visible in all age groups of Sosnowiec inhabitants. It should be mentioned that just before the COVID-19 pandemic, tick-borne Lyme disease was (probably) the most widely recognized viral disease in Poland. During the research, this problem coexisted, especially with the encouragement by various authorities to spend time outside the home, preferably in green areas. This probably explains the convergence of opinions about the threat of ticks among all the surveyed age groups.

It is also interesting that among those city dwellers (approx. 50%) who fear anything, there are attributes related to the presence of specific groups of animals. Another interesting result of the research in this regard is that fears of this type prevailed in men. It is also interesting that over 12% of respondents are afraid of larger mammals (mainly wild boars). Notably, even 30 years ago, for obvious reasons, these species were not recorded in the city, or to a much lesser extent.

It was also interesting that the inhabitants are not afraid of visits to forested post-mining areas. A relatively small percentage of responses noted the threat of crime (12.7% of respondents). Cases of serious crime in the forested post-mining areas have been negligible in the last 20 years, although these crimes have been publicized by the local media each time. The frequency of visits to the forest also influenced the assessment of the threat of potential crimes [χ^2^ (5, N = 300) = 21.7, *p* = 0.00059, ΦC = 0.269]. As mentioned earlier, the general decline in criminal offenses recorded in Sosnowiec is also not without significance with regard to this issue. Meanwhile, phenomena related to former mining operations can be observed (the so-called poor shafts, sinkholes, post-mining waste not fully explored). These issues only appeared in two answers: ‘other’. Frequent visits to the forest also sensitized the respondents to other issues not listed in the previous items [χ^2^ (5, N = 300) = 11.6, *p* = 0.040, ΦC = 0.196]. The notes from the surveys show that these were mainly issues related to littering, illegal landfilling of municipal and construction waste, or ‘unacceptable’ behavior, according to the respondents, mainly concerning young people.

Another important issue that encouraged people to spend time in the studied green areas was their relatively good knowledge of their topography. In each age group, the ignorance of the area was low. This fact was probably due to the dependence on the frequency of visits to forests [χ^2^ (5, N = 300) = 11.9, *p* = 0.036, ΦC = 0.199], which contributed to a better understanding of even larger forest complexes.

An interesting element of the research, also pointing to urban forests’ potential, was that half of the respondents did not see any threats with regard to visiting these areas. The sense of security is enhanced by such factors as information campaigns in the media (ticks, wild boars), the fact that two people, families, or larger groups visit the forest, as is noticeable in our research, and a general decline in common crimes in the city and the region. The following elements were pointed out: the risk of crime, tick bites, an encounter with wild animals (mainly wild boars), not knowing the forest, the distance between the forest and the place of residence being too great, and traffic or industrial noise disturbing relaxation. These are real problems connected with using green areas in Poland. This is also confirmed by the percentage of answers presented in Table 7.

## 5. Discussion

Forest complexes in post-mining areas are among the most characteristic elements of land use in functionally derelict areas. As only some mines were located within cities, forests in post-mining areas within their administrative boundaries are relatively rare. However, the limited nature of mining functions is not the only reason for the rarity of this type of forest within the boundaries of urban centers. The area where they grow is also subject to intra-city competition for space, and in this competition, forests compete with re-industrialization, new municipal or transport investments, and housing. Some mining areas, especially those related to opencast mining, are also used as reservoirs. However, forests in post-mining areas can emerge, as they are often created when cities shrink or functionally disintegrate. In certain circumstances, as examples from eastern Germany show, they can even become a policy objective to offset the consequences of urban shrinkage [5,82].

In the functioning of forests in urban post-mining areas, two groups of conditions—environmental and socio-economic—appear to be crucial.

Environmental conditions are mainly related to surface relief and the degree of degradation of the substrate on which previous mining activities took place. The second environmental issue related to forests in post-mining areas is ecosystem services.

Anthropogenic forests in post-mining cities offer similar ecosystem benefits (ecosystem services) to natural systems [83,84]. These are the benefits that society enjoys from ecosystems, and this concept provides a new way of understanding human–nature relationships [85]. The ecosystem benefits approach supports and facilitates evidence-based decision-making to improve human well-being. For this reason, it has been recognized as a useful approach for various planning practices, including spatial planning [86], planning and managing protected areas [87], environmental damage assessment, environmental impact assessment [72,88], and strategic environmental assessment [89].

Interestingly, research on Sosnowiec shows some differences in the perception of forests in post-mining areas with research results in the Sokolov region in the Czech Republic [90], while in the forests we studied, this attribute was not practically taken into account in assessing a given forest. The differences probably result from the size and type of post-mining forms. These were opencast mines and large heaps in the Czech Republic, whereas in Sosnowiec, they were deep mining and relatively minor post-mining forms. It also seems that in the case of urban forests, where there is a specific deficit of green areas in general (taking into account the population density), each type of green area is a socially expected element of spatial development. On a regional scale, there is a greater possibility of choosing a place of recreation in forest areas.

The research conducted in Sosnowiec, which shows significant acceptance of urban forest in post-mining brownfield sites, also confirms the thesis contained in the research by Rink and Arndt [39]. These authors indicated that in a larger, highly dense city, such as Leipzig, forests of this type constitute a socially expected element of space. However, this does not occur right away. The early succession of trees or afforestation with young trees is less often assessed than forests that have been growing for a long time.

Among the socio-economic determinants, two issues should be highlighted—the long-term nature of the city’s disintegration and the trends in local policy regarding these areas. In terms of urban policy, however, a fundamental problem emerges. The solution to the adverse effects of urban shrinkage can be found by promoting green areas to improve living conditions and sustainable development. Promoting forests in areas degraded and abandoned by industry and mining is becoming an essential element of urban policy in shrinking eastern German cities [91]. Some of the tools and solutions used in revitalizing and developing degraded areas in Sosnowiec (and some other cities) are based on German assumptions [10], and so their partial implementation is possible. There are also some barriers. The policy of creating new green spaces in the city has a broader context: Sosnowiec—Master Plan—Study of the Conditions and Directions for Spatial Development, [64]. Although the city authorities understand this issue, they are criticized in public forums on social media for their defensive policy (www.gazeta.sosnowiec.pl) (accessed on 15 January 2022). However, there is a problem here related to the fact that most forests, including those in cities, belong to a state-owned entity, the State Forests. In the case of cities in post-mining areas, some forests are additionally managed by another state entity, the Mine Restructuring Company.

Consequently, municipal authorities have limited possibilities in terms of managing such forests. This is visible in the case of the mass felling of trees in Sosnowiec (and around the whole of Poland) in areas managed by the State Forests. The municipal authorities are often blamed on social media.

The felling of trees is an activity carried out by the State Forests, whereas all activities related to the urbanization of green areas, the transformation of forests into parks, the over-saturation of green areas with recreational infrastructure, and the lack of response to littering are, in the opinion of residents, heavily attributed to the municipal authorities.

Whatever the urban policy, urban greenfield sites are an essential objective for those interested in residential and economic investment. From the point of view of policies supporting the greening of cities, it may not seem beneficial to promote economic, residential, or municipal investments that compete with green areas.

Attempts to solve this dilemma are already visible in Sosnowiec. These generally consist of allocating a part of already forested post-mining areas for housing and new economic investment purposes (industry, logistics). The most spectacular example is the new housing estate built in the middle of the forest in the post-mining area in the Niwka district. Of course, the discussion on the problem of human–environment conflict is not new. In the context of shrinking post-mining towns in regions such as Silesian Province, it is somewhat necessary to point out the limit of interference with the natural environment because, as another study in this city [13] shows, inhabitants’ opinions on the reforestation/leisure functions and re-industrialization of post-mining brownfield sites are split in half.

Opinions about the existing post-mining forests’ role are more precise, as indicated by the research presented in this article. Discrepancies in opinions concern selected issues and perceptions of post-mining forests, but not the need to increase their availability for the needs of residents in general. This is very important, because there are a dozen or so such cities in southern Poland.

Seen against the background of these fundamental issues based on the human–environment relationship, an issue of social importance is what a forest on a post-mining site in a large post-industrial and, at the same time, shrinking city should be. What the research carried out in Sosnowiec highlights above all is the need for urban greenery. It is of secondary importance for many inhabitants whether this greenery will function as a forest in the urban space. They point more to a park, while other residents prefer a forest. Intense polarization of opinions can even be seen with regard to the idea of not paving one of the roads in Las Zagórski (a former post-mining area) to make it more accessible for cyclists and walkers (www.gazeta.sosnowiec.pl) (accessed on 15 January 2022). So, does this ambiguity of opinion form the social background of a municipal policy trying to reconcile different trends in land use? Undoubtedly, the view that green areas but not necessarily forests are essential for residents broadly supports the regional and national policy guidelines that separate the issue of residents’ accessibility to green areas from the promotion of biodiversity and forests. This accessibility is not necessarily based on the existence of urban forests. An essential element of the discussion about forests in post-mining areas remains the issue of legal protection. Although unique natural values characterize only a small part of the post-mining forests (e.g., the aforementioned protected area ‘Bory’), a few larger forest areas meet the conditions for creating the so-called cultural park. These are Zagórze Forest, the post-mining forest in the Józefów district in the north of the city, Kamionka Forest in the Mec district, the forest in the Dorota district and the forest on the border of Dańdówka and Niwka. This form of protection focuses on the historical economic heritage of the places and their landscape value. Although the name of the forest suggests an attribute of nature, the post-mining note indicates its genesis. Interestingly, the natural attribute has become a valuable factor in preserving cultural and historical heritage.

An essential element of the research was the finding that post-mining forests in a densely populated city are becoming a socially expected form of spatial development. An essential role of post-mining forests in Sosnowiec in the human–environment relationship is that they occupy an area seven times larger than parks. This demonstrates the a significant potential of the formally urban space, in that it could serve the city’s inhabitants more than before. The positive perception of forest areas in Sosnowiec is visible both in the declarations of residents (including half of them who do not see any threats or dangers related to being in forests) and those publicly expressed in social media. Any outside interference is criticized, such as tree felling, littering, illegal waste storage, and allocating forest areas for other purposes. The research also showed a relatively high convergence of views on the post-mining forest among women and men. Differences occurred among respondents only due to their age. However, these differences are apparent, taking into account the way of spending free time and the physical and health condition, differentiating views on the forest and its perception. A significant challenge is the need to pay more attention to these areas by municipal authorities. Although most of these areas are not owned by the Sosnowiec commune, the coordinating role of the municipal authorities in increasing the availability of post-mining forests is very desirable. Some of them even meet the criteria of partial protection as cultural parks. The economic heritage of the area is closely related to the socially needed function of a post-mining urban forest.

## 6. Conclusions

Urban forests are an essential functional and spatial element. However, the social and ecological need for urban forest development sometimes stands in contrast to competing forms of urban development. In this respect, the contrast with various urban forms without woodland (industry, logistics, residential functions) is particularly accentuated. At the same time, the competition for space with parks plays a lesser role. The example of the Polish city of Sosnowiec points to yet another element. The liquidation of the mining industry gave a seemingly great chance for reforestation in the city. However, as it transpires, the deindustrialization of the city is not an obvious direction for its spatial transformation. The competition of the forest for space takes place in conjunction with the urban shrinkage phenomenon, against which solutions are formulated that are not always compatible with reforestation. In such a city, the social and health needs of city dwellers, which are met, among others, by proximity to the forest, contrast with other needs.

However, as the research results show, forests in post-mining areas are an important place for the inhabitants. As many as two-thirds of visitors to forests visit at least once a week. Hence, the frequency of visits is one of the important postulates regarding greater accessibility of individual forest complexes. Accessibility, in turn, is one of the expected aspects of better forest management. This is certainly the most challenging issue, due to the fact that forests are managed by various entities (Sosnowiec commune, State Forests, mine restructuring company, private companies). This issue also relates to the role of biodiversity in post-mining forests. Forest management, especially in terms of the availability of post-mining forests, is important in the context of biodiversity noticed and appreciated by the inhabitants.

The biodiversity appreciated by inhabitants is more valuable for them when they can access it more quickly and experience it without problems. While biodiversity value strongly influences the willingness to spend time in post-mining forests, some pejorative issues were also observed. Among the threats, the inhabitants most often pointed to those related to the local fauna—wild animals and ticks spreading pathogenic viruses. The relatively low share of threats and inconveniences related to the direct participation of people (criminal offenses, devastation, noise, etc.), additionally taking into account the fact that half of the respondents see no threats in forests, undoubtedly creates favorable prospects for the coexistence of post-mining forests and city residents.

## Figures and Tables

**Table 1:** Distribution of the sample of adult residents of Sosnowiec by sex and age (source: the authors, based on the Central Statistical Office Poland, Local Data Bank [65].

Sample Distribution	Female			Male			The Total Population in Particular Groups		
	18–34 Years	35–54 Years	55 Years and Over	18–34 Years	35–54 Years	55 Years and Over	18–34 Years	35–54 Years	55 Years and Over
Population in 2019	17,499	29,348	44,307	18,287	29,481	32,037	35,786	58,829	76,344
Population in 2019 in %	10.24	17.17	25.92	10.7	17.24	18.74	20.93	34.41	44.66
Numerical distribution of the sample after correction	31	52	77	32	52	56	63	104	133

**Table 2:** General features of forest communities in the studied post-mining areas of Sosnowiec (source: the authors).

No.	Type of Vegetation and Woody and Shrubby Species Dominants	Local Names	Forest Layers *	Forest Area in km^2^	Compactness	The Age Structure	Type of (Post) Mining Activity **
1.	Mixed poplar and birch forest: Tree layer (A): *Populus tremula*, *Betula pendula*, *Robinia pseudoacacia* and single *Pinus sylvestris*.	Bergi	A,B,D	0.61	4–5	30–40	1,2,3,4
2.	Fresh pine forest *Leucobryo-Pinetum*: Tree layer (A): *P. sylvestris*, *Q. robur*, *Acer* pseudoplatanus. Shrub layer (B): *Sorbus aucuparia* and *Padus serotina. Vaccinium myrtillus* and *V. vites-idaea.*	Bory	A,B,C,D	0.31	4–5	50–60	4
3.	Artificial mixed forest: Tree layer (A): *R. pseudoacacia*, *B. pendula*, *P. sylvestris*, *Q. rubra.* Shrub layer (B) mostly *Padus serotina*.	Dorota	A,B,C	0.32	4–5	60–70	1,2
4.	Artificial pine plantation: *P. sylvestris* with a single share of *B. pendula* and *Populus tremula*. Shrub layer (B): a single specimen of *P. tremula.*	Hałda Feliks	A,B	0.23	5	20–30	2
5.	Artificial mixed oak-pine-birch forest: Tree layer (A): *Q. rubra*, *Q. robur. P. sylvestris*, *B. pendula*, *R. pseudoacacia*, *Populus alba* and *Padus serotina* in B layer (shrub).	Jęzor Południe	A,B,C	2.13	3–4	60–70	4
6.	Deciduous mixed forests *Robinia-Populus-Betula-Acer:* Tree layer (A): *R. pseudocacia*, *Populus tremula*, *Betula pendula*, *A. platanoides*, *A. pseudoplatanus*, *Tilia cordata*, *A. negundo*. Shrub layer: *Sambucus nigra*, *S. aucuparia*, *Corylus avellana*, *Prunus serotina*, *Crataegus monogyna*.	Józefów	A,B,C,D	0.38	5	70–80	1,2,3
7.	Mixed forests with black locust and maple forest: Tree layer: R. pseudoacacia, *A. platanoides*, *A. pseudoplatanus*, *A. negundo*, *Tilia cordata*, *Ulmus montana*, *U. minor* and *Populus nigra.* Shrub layer: *Caragana arborescens Juglans regia*, *Malus domestica.*	Kamionka—Dańdówka	A,B,C,D	0.03	3–4	50–60	5
8.	Elm and oak forest: Tree layer: *Ulmus laevis*, *Quercus robur*, *Carpinus betulus*, *A. platanoides*, *A. pseudoplatanus*, *Betula obscura*, *Aesculus hipocastanum*. Shrub layer (B): *Corylus avellana*, *Prunus serotina*, *Crataegus monogyna*, *Cornus sanguinea*, *Sambucius nigra*.	Kamionka—Mec	A,B,C,D	0.06	5	40–50	5
9.	Artificial birch forest: Mainly *B. pendula* in tree layer. B layer: *Padus serotina* and *Hippophae rhamnoides.*	Klimontów (Mine)	A,B	0.35	5	30	2
10.	Artificial mixed forest: Tree layer: *Q. rubra*, *R. pseuduacacia*, *Fagus sylvatica*, *A. negundo*, *P. sylvestris*, *P. nigra*, *P. alba.* Shrub layer: *Caragana arborescens*, *Salix caprea*, *S. pupurea*.	Las Zagórski	A,B,C,D	1.93	4–5	60–70	1,2,3
11.	Artificial initial pine forest: Tree layer: *P. sylvestris*, *Q. robur* individually as an admixture, *Acer pseudoplatanus*, Shrub layer: *Sorbus aucuparia*, *Padus serotina* and tree growths.	Lasek	A,B,D	0.64	5	50–60	1,2,3
12.	Artificial mixed forest: Tree layer: *A. platanoides*, *A. pseudoplatanus B. pendula*, *Q. robur*, *Q. rubra*, *Pinus sylvestris*, *R. pseudoacacia*, *P. tremula*, *U. leavis.* B layer: *Caragana arborescens*, *Hippophae rhamnoides*, *Padus avium*, *P. serotina*, *Sambucus nigra*, *Symphoricarpos albus*, *Spiraea salicifolia.*	Milowice	A,B,C	0.52	3,4–5	60–70	1,2,4
13.	Initial stage of *Leucobryo-Pinetum*: Tree layer: *P. sylvestris*, *Q. robur*, *B. pendula*, *P. tremula*, *R. pseudoacacia.* Srub layer (B): *P. serotina*, *Juniperus communis*, *S. aucuparia* and tree growths.	Rybaczówka	A,B,C	0.76	4–5	50–60	4

Explanations: * Forest Layers: A—tree layer; B—shrub layer; C—undergrowth; D—forest floor (mainly plant litter). ** type of (post) mining activity: 1—dumps of post-mining (coal) waste; 2—shafts and areas of hard coal mines; 3—illegal small, old mine shafts; 4—sand pits; 5—limestone or dolomite mines.

**Table 3:** Respondents’ answers (by sex and age) to the question: how often do you visit a forest or a park in the city of Sosnowiec? (Source: the authors).

Answers	F 18–34 Years		F 35–54 Years		F 55 Years and Over		M 18–34 Years		M 35–54 Years		M 55 Years and Over		Total
	N.P.	%	N.P.	%	N.P.	%	N.P.	%	N.P.	%	N.P.	%	N.P.
Every day	0	0.0	4	1.3	14	4.7	6	2.0	8	2.7	8	2.7	40
Once a week	22	7.3	29	9.7	35	11.7	17	5.7	22	7.3	34	11.3	159
Once a month	7	2.3	12	4.0	17	5.7	8	2.7	17	5.7	11	3.7	72
Once a year	2	0.7	5	1.7	6	2.0	1	0.3	4	1.3	2	0.7	20
Every few years	0	0.0	0	0.0	0	0.0	0	0.0	0	0.0	1	0.3	1
I do not	0	0.0	2	0.7	5	1.7	0	0.0	1	0.3	0	0.0	8
Total	31	10.3	52	17.3	77	25.7	32	10.7	52	17.3	56	18.7	300

Explanations: N.P.—the number of persons; F—female; M—male.

**Table 4:** Respondents’ answers (by sex and age) to the question: should a green area in your area be a more accessible forest or park? (Source: the authors).

Answers	F 18–34 Years		F 35–54 Years		F 55 Years and Over		M 18–34 Years		M 35–54 Years		M 55 Years and Over		Total
	N.P.	%	N.P.	%	N.P.	%	N.P.	%	N.P.	%	N.P.	%	N.P.
Yes	20	6.7	37	12.3	46	15.3	22	7.3	30	10.0	34	11.3	189
No	10	3.3	15	5.0	25	8.3	8	2.7	19	6.3	17	5.7	94
No opinion	1	0.3	0	0.0	6	2.0	2	9.3	3	1.0	5	1.7	17
Total	31	10.3	52	17.3	77	25.7	32	10.7	52	17.3	56	18.7	300

Legend: N.P.—the number of persons; F—female; M—male.

**Table 5:** Respondents’ answers (by sex and age) to the question: should this area be better managed (alleys, lighting, recreation)? (Source: the authors).

Answers	F 18–34 Years		F 35–54 Years		F 55 Years and Over		M 18–34 Years		M 35–54 Years		M 55 Years and Over		Total
	N.P.	%	N.P.	%	N.P.	%	N.P.	%	N.P.	%	N.P.	%	N.P.
Forest	8	2.7	13	4.3	35	11.7	14	4.7	16	5.3	19	6.3	105
Park	22	7.3	36	12.0	40	13.3	15	5.0	30	10.0	31	10.3	174
No opinion	1	0.3	3	1.0	2	0.7	3	1.0	6	2.0	6	2.0	21
Total	31	10.3	52	17.3	77	25.7	32	10.7	52	17.3	56	18.7	300

Legend: N.P.—the number of persons; F—female; M—male.

**Table 6:** Respondents’ answers (by sex and age) to the question: do you appreciate this area’s natural diversity? (Source: the authors).

Answers	F 18–34 Years		F 35–54 Years		F 55 Years and Over		M 18–34 Years		M 35–54 Years		M 55 Years and Over		Total
	N.P.	%	N.P.	%	N.P.	%	N.P.	%	N.P.	%	N.P.	%	N.P.
Yes	25	8.3	41	13.7	68	22.7	25	8.3	40	13.3	49	16.3	248
No	2	0.7	9	3.0	4	1.3	3	1.0	6	2.0	6	2.0	30
No opinion	4	1.3	2	0.7	5	1.7	4	1.3	6	2.0	1	0.3	22
Total	31	10.3	52	17.3	77	25.7	32	10.7	52	17.3	56	18.7	300

Legend: N.P.—the number of persons; F—female; M—male.

**Table 7:** Respondents’ answers (by sex and age) to the question: what deters you from wandering in a forest or park in the city? (Source: the authors).

Answers	F 18–34 Years		F 35–54 Years		F 55 Years and Over		M 18–34 Years		M 35–54 Years		M 55 Years and Over		Total
	Y%	N%	Y%	N%	Y%	N%	Y%	N%	Y%	N%	Y%	N%	Y%
The threat of crime	1.7	8.7	1.3	16.0	3.7	22.0	2.3	8.3	1.0	16.3	2.7	16.0	12.7
Ticks	2.3	8.0	2.0	15.3	8.0	17.7	3.0	7.7	3.7	13.7	5.7	13.0	24.7
Wild animals	1.3	9.0	0.7	16.7	3.7	22.0	1.3	9.3	1.7	15.7	3.7	15.0	12.3
Being unacquainted with the area	1.3	9.0	1.0	16.3	3.0	22.7	1.7	9.0	1.3	16.0	2.7	16.0	11.0
Distance from home	1.7	8.7	2.3	15.0	3.7	22.0	1.3	9.3	2.3	15.0	3.0	15.7	14.3
Noise	0.0	10.3	0.7	16.7	1.0	24.7	0.3	10.3	1.3	16.0	0.3	18.3	3.7
Other	0.3	10.0	2.3	15.0	0.7	25.0	0.3	10.3	2.0	15.3	1.0	17.7	6.7
Nothing deters me	4.7	5.7	10.0	7.3	11.0	14.7	5.7	5.0	8.3	9.0	8.7	10.0	48.3

Legend: F—female; M—male; Y—yes: N—no.

## Data Availability

Not applicable.

## Appendix A. Survey Developed with the CATI Method for Forest Areas

**Information about Respondents**	
**Question**	**Response**
Sex:	□ Female □ Male
Age:	□ 18–34 years □ 35–54 years □ 55 years and over
Neighbourhood:	□ Bór □ Dańdówka □ Dębowa Góra □ Klimontów □ Maczki □ Mec □ Milowice □ Niwka □ Osiedle Juliusz □ Osiedle Piastów □ Osiedle Stulecia □ Ostrowy Górnicze □ Pekin □ Pogoń □ Porąbka □ Rabka □ Sielec □ Stary Sosnowiec □ Środula □ Śródmieście □ Zagórze □ Other ...............................................
**Information on forest areas**	
1. How often do you visit a forest or a park in the city of Sosnowiec?	□ 1. Every day □ 2. Once a week □ 3. Once a month □ 4. Once a year □ 5. Every few years □ 6. I do not
2. Should a green area in your area be a more accessible forest or park?	□ 1. Forest □ 2. Park □ 3. No opinion
3. Should this area be better managed (alleys, lighting, recreation)?	□ 1. Yes □ 2. No □ 3. No opinion
4. Do you appreciate this area’s natural Diversity?	□ 1. Yes □ 2. No □ 3. No opinion
5. What deters you from wandering in a forest or park in the city?	□ 5.01. The threat of crime □ 5.02. Ticks □ 5.03. Wild animals □ 5.04. Being unacquainted with the area □ 5.05. Distance from home □ 5.06. Noise □ 5.07. Other ............................................... □ 5.08. Nothing deters me

## Appendix B. Results of the Chi-Square Test of Independence for Features from the Survey Developed with the CATI Method for Forest Areas

**Statistic**	**Statistics: 1. How Often Do You Visit a Forest or a Park in the City of Sosnowiec? (6) × 2. Should a Green Area in Your Area Be a More Accessible Forest or Park? (3) (BASE)**		
	**Chi-Square**	**df**	***p***
Pearson Chi-square	37.47531	df = 10	*p* = 0.00005
M-L Chi-square	28.31869	df = 10	*p* = 0.00160
Phi	0.3534370		
Contingency coefficient	0.3332358		
Cramér’s V	0.2499177		

**1. How Often Do You Visit a Forest or a Park in the City of Sosnowiec?**	**Two-Way Summary Table: Observed Frequencies (Base in BASE) Marked Cells Have Counts > 4**			
	**2. Should a Green Area in Your Area Be a More Accessible Forest or Park.** **2. Park**	**2. Should a Green Area in Your Area Be a More Accessible Forest or Park** **1. Forest**	**2. Should a Green Area in Your Area Be a More Accessible Forest or Park** **3. No Opinion**	**Row** **Totals**
2. Once a week	103	51	5	159
Column %	59.20%	48.57%	23.81%	
3. Once a month	40	22	10	72
Column %	22.99%	20.95%	47.62%	
4. Once a year	11	6	3	20
Column %	6.32%	5.71%	14.29%	
1. Every day	15	24	1	40
Column %	8.62%	22.86%	4.76%	
6. I do not	5	2	1	8
Column %	2.87%	1.90%	4,76%	
5. Every few years	0	0	1	1
Column %	0.00%	0.00%	4.76%	
Totals	174	105	21	300

**Statistic**	**Statistics: 1. How Often Do You Visit a Forest or a Park in the City of Sosnowiec? (6) × 3. Should This Area Be Better Managed (Alleys, Lighting, Recreation)? (3) (BASE)**		
	**Chi-Square**	**df**	***p***
Pearson Chi-square	31.06643	df = 10	*p* = 0.00057
M-L Chi-square	18.39142	df = 10	*p* = 0.04871
Phi	0.322337		
Contingency coefficient	0.306793		
Cramér’s V	0.227927		

**1. How Often Do You Visit a Forest or a Park in the City of Sosnowiec?**	**Two-Way Summary Table: Observed Frequencies (Base in BASE) Marked Cells Have Counts > 4**			
	**3. Should This Area Be Better Managed (Alleys, Lighting, Recreation)?** **2. No**	**3. Should This Area Be Better Managed (Alleys, Lighting, Recreation)?** **1. Yes**	**3. Should This Area Be Better Managed (Alleys, Lighting, Recreation)?** **3. No Opinion**	**Row** **Totals**
2. Once a week	49	102	8	159
Column %	52.13%	53.97%	47.06%	
3. Once a month	24	44	4	72
Column %	25.53%	23.28%	23.53%	
4. Once a year	6	10	4	20
Column %	6.38%	5.29%	23.53%	
1. Every day	11	29	0	40
Column %	11.70%	15.34%	0.00%	
6. I do not	4	4	0	8
Column %	4.26%	2.12%	0.00%	
5. Every few years	0	0	1	1
Column %	0.00%	0.00%	5.88%	
Totals	94	189	17	300

**Statistic**	**Statistics: 1. How Often Do You Visit a Forest or a Park in the City of Sosnowiec? (6) × 4. Do You Appreciate This Area’s Natural Diversity? (3) (BASE)**		
	**Chi-Square**	**df**	***p***
Pearson Chi-square	42.62672	df = 10	*p* = 0.00001
M-L Chi-square	30.60505	df = 10	*p* = 0.00068
Phi	0.377577		
Contingency coefficient	0.353236		
Cramér’s V	0.266987		

**1. How Often Do You Visit a Forest or a Park in the City of Sosnowiec?**	**Two-Way Summary Table: Observed Frequencies (Base in BASE) Marked Cells Have Counts > 4**			
	**4. Do You Appreciate This Area’s Natural Diversity?** **1. Yes**	**4. Do You Appreciate This Area’s Natural Diversity?** **3. No Opinion**	**4. Do You Appreciate This Area’s Natural Diversity?** **2. No**	**Row** **Totals**
2. Once a week	138	6	15	159
Column %	55.65%	27.27%	50.00%	
3. Once a month	59	8	5	72
Column %	23.79%	36.36%	16.67%	
4. Once a year	10	6	4	20
Column %	4.03%	27.27%	13.33%	
1. Every day	36	0	4	40
Column %	14.52%	0.00%	13.33%	
6. I do not	5	1	2	8
Column %	2.02%	4.55%	6.67%	
5. Every few years	0	1	0	1
Column %	0.00%	0.00%	0.00%	
Totals	248	22	30	300

**Statistic**	**Statistics: M4. Age (3) × 5.02. pw. Ticks (2) (Base in BASE)**		
	**Chi-Square**	**df**	***p***
Pearson Chi-square	6.609019	df = 2	*p* = 0.03672
M-L Chi-square	6.840868	df = 2	*p* = 0.03270
Phi	0.148425		
Contingency coefficient	0.146817		
Cramér’s V	0.148425		

**M4. Age**	**Two-Way Summary Table: Observed Frequencies (Base in BASE) Marked Cells Have Counts > 4**		
	**5.02. pw. Ticks** **0**	**5.02. pw. Ticks** **1**	**Row** **Totals**
3. 55 years and over	92	41	133
Column %	40.71%	55.41%	
1. 18–34 years	47	16	63
Column %	20.80%	21.62%	
2. 35–54 years	87	17	104
Column %	38.50%	22.97%	
Totals	226	74	300

**Statistic**	**Statistics: 1. How Often Do You Visit a Forest or a Park in the City of Sosnowiec? (6) × 5.04. pw. Being Unacquainted with the Area (2) (BASE)**		
	**Chi-Square**	**df**	***p***
Pearson Chi-square	11.90814	df = 5	*p* = 0.03607
M-L Chi-square	8.645664	df = 5	*p* = 0.12406
Phi	0.199233		
Contingency coefficient	0.195393		
Cramér’s V	0.199233		

**1. How Often Do You Visit a Forest or a Park in the City of Sosnowiec?**	**Two-Way Summary Table: Observed Frequencies (BASE) Marked Cells Have Counts > 5**		
	**5.04. pw. Being Unacquainted with the Area** **0**	**5.04. pw. Being Unacquainted with the Area** **1**	**Row** **Totals**
2. Once a week	138	21	159
Column %	51.69%	63.64%	
3. Once a month	68	4	72
Column %	25.47%	12.12%	
4. Once a year	17	3	20
Column %	6.37%	9.09%	
1. Every day	37	3	40
Column %	13.86%	9.09%	
6. I do not	7	1	8
Column %	2.62%	3.03%	
5. Every few years	0	1	1
Column %	0.00%	3.03%	
Totals	267	33	300

**Statistic**	**Statistics: 1. How Often Do You Visit a Forest or a Park in the City of Sosnowiec (6) × 5.01. pw? The Threat of Crime (2) (BASE)**		
	**Chi-Square**	**df**	***p***
Pearson Chi-square	21.70901	df = 5	*p* = 0.00059
M-L Chi-square	17.77878	df = 5	*p* = 0.00324
Phi	0.269004		
Contingency coefficient	0.25977		
Cramér’s V	0.269004		

**1. How Often Do You Visit a Forest or a Park in the City of Sosnowiec?**	**Two-Way Summary Table: Observed Frequencies (BASE) Marked Cells Have Counts > 5**		
	**5.01. pw. The Threat of Crime** **0**	**5.01. pw. The Threat of Crime** **1**	**Row** **Totals**
2. Once a week	142	17	159
Column %	54.20%	44.74%	
3. Once a month	67	5	72
Column %	25.57%	13.16%	
4. Once a year	17	3	20
Column %	6.49%	7.89%	
1. Every day	28	12	40
Column %	10.69%	31.58%	
6. I do not	8	0	8
Column %	3.05%	0.00%	
5. Every few years	0	1	1
Column %	0.00%	2.63%	
Totals	262	38	300

**Statistic**	**Statistics: 1. How Often Do You Visit a Forest or a Park in the City of Sosnowiec? (6) × 5.07. pw. Other? (2) (BASE)**		
	**Chi-Square**	**df**	***p***
Pearson Chi-square	11.62483	df = 5	*p* = 0.04031
M-L Chi-square	8.524585	df = 5	*p* = 0.12960
Phi	0.196849		
Contingency coefficient	0.193142		
Cramér’s V	0.196849		

**1. How Often Do You Visit a Forest or a Park in the City of Sosnowiec?**	**Two-Way Summary Table: Observed Frequencies (BASE) Marked Cells Have Counts > 5**		
	**5.07. pw. Other?** **0**	**5.07. pw. Other?** **1**	**Row** **Totals**
2. Once a week	150	9	159
Column %	53.57%	45.00%	
3. Once a month	68	4	72
Column %	24.29%	20.00%	
4. Once a year	16	4	20
Column %	5.71%	20.00%	
1. Every day	39	1	40
Column %	13.93%	5.00%	
6. I do not	6	2	8
Column %	2.14%	10.00%	
5. Every few years	1	0	1
Column %	0.36%	0.00%	
Totals	280	20	300
